# More Than Telemonitoring: Health Provider Use and Nonuse of Life-Log Data in Irritable Bowel Syndrome and Weight Management

**DOI:** 10.2196/jmir.4364

**Published:** 2015-08-21

**Authors:** Chia-Fang Chung, Jonathan Cook, Elizabeth Bales, Jasmine Zia, Sean A Munson

**Affiliations:** ^1^ Department of Human Centered Design & Engineering Dub Group University of Washington Seattle, WA United States; ^2^ Division of Design Dub Group University of Washington Seattle, WA United States; ^3^ Department of Computer Science & Engineering Dub Group University of Washington Seattle, WA United States; ^4^ Department of Medicine Division of Gastroenterology University of Washington Seattle, WA United States

**Keywords:** life logs, behavioral self-monitoring, clinical care, chronic disease, health, wellness, personal informatics, quantified self

## Abstract

**Background:**

The quantified self, self-monitoring or life-logging movement is a trend to incorporate technology into data acquisition on aspects of a person's daily life in terms of inputs (eg food consumed), states (eg mood), and performance (mental and physical). Consumer self-monitoring mobile phone apps have been widely studied and used to promote healthy behavior changes. Data collected through life-logging apps also have the potential to support clinical care.

**Objective:**

We sought to develop an in-depth understanding of providers’ facilitators and barriers to successfully integrating life-log data into their practices and creating better experiences. We specifically investigated three research questions: How do providers currently use patient-collected life-log data in clinical practice? What are provider concerns and needs with respect to this data? What are the constraints for providers to integrate this type of data into their workflows?

**Methods:**

We interviewed 21 health care providers—physicians, dietitians, a nurse practitioner, and a behavioral psychologist—who work with obese and irritable bowel syndrome patients. We transcribed and analyzed interviews according to thematic analysis and an affinity diagramming process.

**Results:**

Providers reported using self-monitoring data to enhance provider-patient communication, develop personalized treatment plans, and to motivate and educate patients, in addition to using them as diagnostic and adherence tools. However, limitations associated with current systems and workflows create barriers to regular and effective review of this data. These barriers include a lack of time to review detailed records, questions about providers' expertise to review it, and skepticism about additional benefits offered by reviewing data. Current self-monitoring tools also often lack flexibility, standardized formats, and mechanisms to share data with providers.

**Conclusions:**

Variations in provider needs affect tracking and reviewing needs. Systems to support diagnosis might require better reliability and resolution, while systems to support interaction should support collaborative reflection and communication. Automatic synthesis of data logs could help providers focus on educational goals while communication of contextual information might help providers better understand patient values. We also discuss how current mobile apps and provider systems do, and do not, support these goals, and future design opportunities to realize the potential benefits of using life-logging tools in clinical care.

## Introduction

### Overview

People increasingly turn to mobile phone apps and wearable devices for health management. Nearly 70% of US adults track at least one health indicator for themselves or for a loved one, especially if they suffer from a chronic medical condition; 21% use some form of technology to do so [1]. This year, an estimated 500 million people worldwide will use a health care app [2]. There are already over 5000 self-monitoring apps in Apple’s iOS app store alone, including physical activity (eg, Moves [3]), sleep (eg, Sleep Cycle [4]), and food journaling (eg, MyFitnessPal [5]) apps.

Many companies promise to take advantage of cheaper, low-power sensors and ubiquitous connectivity to make patient data about everyday health factors, behaviors, and outcomes available to their health providers. This, they argue, will lead to higher quality and less expensive health care. Partnerships between consumer device manufacturers and health care systems, such as Apple’s HealthKit [6] in which Apple collaborated with hospital systems and electronic health record (EHR) vendors, also promise to make the vast array of data collected from these apps available to health providers. To achieve this potential, designers and administrators must consider provider goals, perceptions, and workflows.

### Health Information Technology and Life Logs

Self-monitoring—using data manually entered by individuals or objectively collected with sensors in phones or other devices—has proven valuable for patients managing chronic diseases that require behavior change [7,8]. Prior work has identified barriers to using nonelectronic tracking tools, such as paper-based diaries, for managing chronic diseases, either individually or in consultation with providers [9,10]. Electronic diaries have resulted in improved compliance rates, more complete and higher quality entries, and greater user satisfaction in comparison to paper diaries [9,11,12]. Wearable devices and mobile phone apps that enable objective monitoring, such as wirelessly connected pedometers, can further reduce user burdens and improve the completeness and precision of data.

Consumer-focused life logs are a commonly used type of self-monitoring app; these may contain health and wellness data, such as food, physical activity, and mood, as well as nonhealth data, such as location and calendar information. Early life-logging prototypes were envisioned as systems that would capture every aspect of someone’s everyday life [13]. The consumer life-logging and personal informatics apps [14] on today’s market typically track one or two types of data (eg, the mobile phone app, Moves, records locations, how people travel between those locations, and physical activity) or are designed to help people inspect and manage a particular health concern or other aspect of their life. Mobile apps that track physical activity and increase user awareness of activity levels can increase motivation for behavior change [15,16]. Other systems have been designed to help end users identify factors that influence their wellness behaviors and outcomes, for example, as discussed in Bentley et al [17] and Kay et al [18]. These life-logging and tracking tools have the potential to make continuous, objective, and precise data available for clinical care. Integration of data that many people already collect using these apps may then enrich medical care without adding new burdens for patients.

While there is increasing interest in incorporating these data into electronic medical records [19], how to integrate these data into provider workflows without increasing provider burdens remains underexplored.

### Provider-Patient Communication

Technology tools can also enhance patient-provider communication. Better communication in medical care correlates with better patient adherence [20] and other intermediate outcomes associated with improved health [21]. Many studies have identified important objectives of provider-patient communication, such as creating a good interpersonal relationship, exchanging information, and performing shared decision making [22]. Technology tools can support these objectives. Studies of patient-care management tools for cancer [23] and diabetes [24,25] have shown how technology can support communication around care. In these studies, symptom-tracking data helped patients promote conversations with clinicians and empowered patients to control their ways of interacting with doctors.

The use of technology during clinical visits can also harm provider-patient communication. While paper-based medical records can provide flexibility and a focus point during clinical conversations, the use of computer-based electronic medical records can create barriers to eye contact and communication [26-28].

In recent years, telemonitoring has shown promise for various health management programs, such as reducing readmission rates for heart failure patients [29,30] and for diabetes management [31,32]. However, most telemonitoring systems have been designed with providers as the primary users and designers have overlooked patient roles in providing context and interpreting symptom data [33].

Self-trackers, on the other hand, predominantly use one or more of a variety of consumer-focused life-logging apps and devices available in the marketplace; these have been designed with the self-tracker as the primary user, independent of medical professionals or other experts. Despite these tools not being designed for the patient-provider interaction, around one-third of current self-trackers have shared data from these apps with their health providers [34]. When shared, however, providers rarely engage with this data, which frustrates many of those self-trackers [34]. These aspirations and frustrations are likely to increase as mobile phones gain more sensors, and more people adopt wearable devices and health tracking apps [1,34]. Integrating data from these apps into clinical care may greatly expand the use of self-monitoring data by medical teams, but at the expense of disrupting current practices and routines.

### Research Goals

#### Overview

In our study, we sought to build upon previous findings to identify specific opportunities and barriers for the use of personal informatics tools in clinical care. We focused on two chronic conditions that are affected by everyday choices: weight management and irritable bowel syndrome (IBS). Over time, both conditions result in high direct and indirect health care costs.

#### Overweight and Obesity

The prevalence of overweight and obesity are increasing in the United States [35] and worldwide [36]. Overweight and obesity are associated with increased risk of cardiovascular diseases, diabetes, hypertension, certain cancers, respiratory problems, and osteoarthritis [37].

Addressing barriers to change, self-monitoring and strategizing how to maintain lifestyle changes have proven to be effective techniques for weight management. Counseling about changes in diet and physical activity is desirable to patients [38], can promote increases in physical activity [39], and results in significant, sustained weight loss [8]. Despite ample evidence supporting behavior change programs in treating obesity, primary care providers report that inadequate training and lack of time are significant barriers to providing counseling for weight-loss patients [10].

To address the resource intensity of in-person behavioral weight-loss programs, technology-enabled approaches are increasingly common. Mobile phone apps that assist with goal setting and self-monitoring may help overweight and obese patients lose weight [12]. Integration of this consumer-collected data with health care providers’ routines may increase and reinforce the efficacy of behavior change efforts, but design and treatment practices to support use of this data are currently unknown.

#### Irritable Bowel Syndrome

IBS is a chronic functional disorder characterized by the presence of episodic abdominal pain associated with diarrhea and/or constipation. It affects up to 20% of the US population [40]. Each individual patient with IBS has a different set of potential behavioral factors that can trigger a symptom flare-up. Providers currently attempt to identify these individualized IBS symptom triggers by manually scanning paper diaries for food, sleep, activity, and symptom correlations. However, these diaries are typically handwritten with incomplete, disorganized, and unreliable data. Therefore, providers often do not have the time or ability to interpret data from patient diaries [11]. As a result, 62.5% of IBS patients are dissatisfied with the feedback they receive from providers regarding their diaries [41].

IBS patients and their providers need a more efficient and effective way to use the data in these diaries to identify individualized lifestyle modifications that result in bowel symptom reduction and improved quality of life. Limited research has been conducted on the perspectives of IBS providers on the goals for collecting, sharing, and representing life-log data.

Through interviews with 21 health care providers, including physicians, nurses, and dietitians, we contribute an understanding of providers' current practices and constraints for reviewing life-log data and their concerns and needs with respect to this data. Most providers already have, and believe they should have, a role in reviewing patients' life-log data; however, their opinions differ on specific roles and how much this review can support diagnosis, development of treatment plans, and the patient-provider relationship. Providers encounter this data when patients bring in their own journals, unsolicited, or after directing patients to track as part of the diagnosis and treatment process.

While many health providers recognize that patients prefer to use, or already use, consumer-focused life-log apps, these apps offer insufficient flexibility to meet provider needs and do not support collaborative use during patient visits. Consequently, providers rarely review data from patient apps, frequently delegate the review of data to others, and usually ask that patients use provider-preferred tools even if they are less convenient for patients. Finally, though it was not the focus of this study, we identified organizational and policy barriers to provider-preferred workflows for reviewing patient-collected data.

## Methods

### Overview

To answer these questions, we conducted interviews with medical providers. Our interviews covered their current use of patient-collected data, how they aspire to use this data (if they do), and what facilitates and inhibits such uses.

### Participants

We recruited 21 primary care providers through word-of-mouth recruitment with colleagues. We focused on health providers in a large, university-affiliated health system (1). To gather perspectives from providers in other health systems, we also interviewed providers in a second university-affiliated health system (2), a health maintenance organization (1), and one independent dietitian. Many providers also had experience working in other university-affiliated health systems (participant IDs: D02, GM09, GM10, D13, D15, GM21, D17, FM18), another health maintenance organization (2) (participant ID: FM16), and other private practices (participant IDs: D02, FM05, FM06, D11, D12). Some of these organizations were in other states (participant IDs: D02, GM09, GM10, D15, FM16, FM18, D20). Participants included 6 family medicine physicians, 1 behavioral psychologist, 1 nurse practitioner, 5 gastroenterologists, and 7 dietitians (see Table 1). We compensated each participant with a US $30 gift card.

**Table 1:** Study participants.

Affiliation	Specialty	Participant IDs
University-affiliated health system (1)	Family medicine physician	FM01, FM05, FM06, FM07
	Dietitian	D02, D11, D12, D13, D15
	Gastroenterologist	GM09, GM10, GM14, GM19
	Behavioral psychologist	BP03
	Nurse practitioner	NP04
	Health navigator	HN08
University-affiliated health system (2)	Dietitian	D20
Health maintenance organization (1)	Family medicine physician	FM16, FM18
	Gastroenterologist	GM21
Independent	Dietitian	D17

The family medicine physicians, dietitians, nurse, and behavioral psychologist we interviewed work with patients on a variety of concerns, including IBS and obesity/overweight, while the gastroenterologists work specifically with patients with digestive problems, such as IBS.

Our results describe practices, goals, and barriers experienced by a variety of health providers. We believe the results describe most US health systems, though we note where we identified differences between health systems. Further, because the providers we interviewed practice at a variety of clinic sites, we were able to learn about experiences providing care to patients with diverse backgrounds and socioeconomic statuses. This is important, as personal informatics tools are commonly critiqued, for the most part, as tools for technically savvy, well-off individuals.

### Interviews

We conducted an hour-long (range 50-70 minutes) semistructured interview with each participant. We interviewed 10 participants in person and 11 by phone. The interview consisted of three segments intended to help us learn about provider experiences, goals, and concerns about using patient-collected life-log data during patient visits (see Multimedia Appendix 1). In the first portion, we asked whether providers currently review any patient-collected life-log data as part of patient visits. If they did, we probed for the type of data, the clinical conditions for which they use this data, the review process, and their best and worst experiences with the review process. If they did not review patient-collected life-log data, we explored why they did not use this data. Next, we followed up with questions exploring how patient-collected life-log data does and does not currently fit into provider workflows. These questions included inquiries into the benefits that the data and its review offers to the provider and their patients, challenges in reviewing data, provider goals in reviewing the data, and roles in the collection and review process.

**Figure 1:**
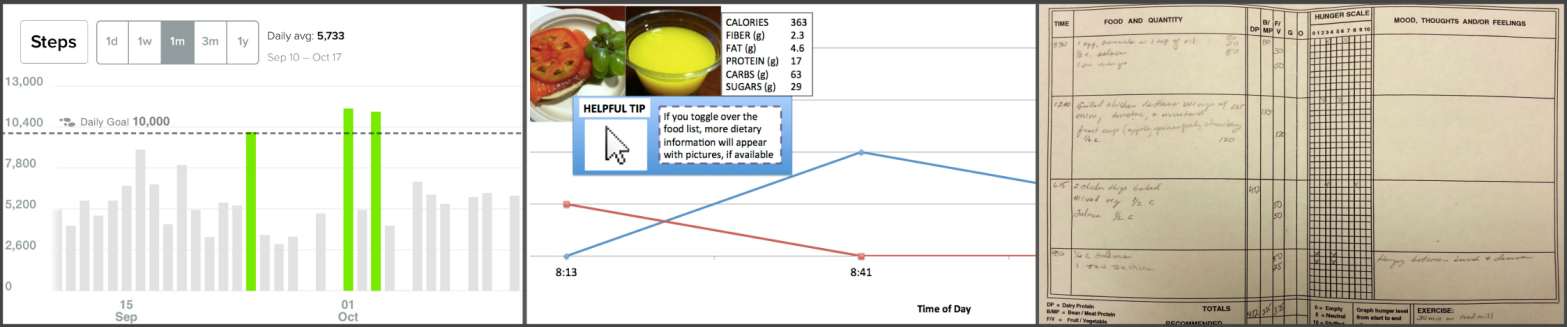
Patient-collected life-log apps (a, b) and a paper-based journal (c).

To help providers react to specific examples of different types of data, including providers who were less familiar with personal informatics tools, we used three paper prototypes in the interviews. These included a dashboard for a physical activity tracking device currently on the market (Fitbit [42]), a mobile app to help IBS patients track symptoms and triggers (Gut Guru [43]), and Health Report [44], a conceptual app that allows patients to track symptoms between visits and then summarize their data before a clinic visit (see Figure 1, a and b). For remote interviews, we presented prototypes using video chat features or sent screenshots by email.

### Analysis Techniques

We audiotaped and transcribed all interviews. The full research team conducted an affinity diagram analysis [45] to identify key themes. We transformed the interview transcripts into approximately 700 affinity notes. After several passes inductively organizing these notes into categories, we identified several themes regarding provider-perceived benefits and barriers to use of consumer-oriented, self-monitoring data (see Multimedia Appendix 2).

In addition to our affinity analysis, we coded each transcript through a mix of deductive (based on our research questions and themes identified in prior work) and inductive coding to capture other emergent themes. Two researchers independently coded the same transcripts and met to resolve ambiguities in the codebook and to add and refine codes reflecting emergent themes. After coding the remaining transcripts, we rereviewed all transcripts to reflect the final codebook. In coding transcripts, we coded especially for goals and barriers to life-log data use.

## Results

### Overview

Overall, providers saw potential benefits for using life-log data, but rarely engaged with data from the apps many patients already use. Of the various types of everyday life-log data, providers were most familiar with symptom and performance diaries, such as an IBS symptom diary or a weight record. Most providers (except participants N04, HN08) were familiar with various forms of food journals. Some (all dietitians and participants BP03, FM01, FM05, FM16, FM17, GM18) had experiences with encouraging patients to track their physical activities or with patients bringing in their physical activity records. Providers also mentioned using or encountering other types of life logs, such as sleep logs (participants FM01, BP03, D12), stress logs (participants FM01, BP03, GM09, D15, FM16, GM18), or mood diaries (participants BP03, D15, D17). MyFitnessPal, Weight Watcher*s* [46], and Fitbit were the most common apps that patients had asked providers to look at and review. Table 2 shows a summary of provider attitudes about the benefits and barriers of self-tracking and reviewing tracked data.

Providers noted that even patients tracking on their own—without review by providers—can be beneficial, but that their instruction and review can help patients overcome many obstacles to effective self-tracking. Providers also reported ways that their review of patient-collected data can help achieve their treatment and communication goals. Despite the many benefits that providers have experienced and perceived, barriers to integrating this data into their workflow remain, and these barriers deter them from fully adopting the practice.

**Table 2:** Summary of results showing provider attitudes about benefits and barriers of self-tracking and reviewing tracked data.

Tracking by patient or provider	Benefits	Barriers and concerns
Patients tracking without provider involvement	Patients can learn about their behaviors, symptoms, progress, and health outcomes. Patients can identify trends and correlations from their data. Patients can become more independent in managing their conditions.	Self-tracking requires extensive patient time and commitment. Patients may not understand what to track and how to track well. Patients may have unrealistic expectations and lose motivation when they cannot immediately reap the benefits of tracking.
Tracking directed by medical team	Tracking overcomes some patient motivational barriers. Tracking provides opportunities for patient education.	Asking patients to track without having providers review the data can send mixed messages. Some providers doubt their ability to advise on tracking; many providers doubt patient ability to review tracking data. Providers may be unfamiliar with currently available tracking tools.
Tracking reviewed by health care providers	Tracking supports diagnosis. Tracking helps personalize treatment(s). Tracking increases patient motivation and accountability. Tracking supports the patient-provider relationship.	Providers have constrained time. Providers question additional health benefits from provider review of tracked data. There is a lack of tracking tool flexibility. There is a lack of tracking tool standardization. There are no established mechanisms for patients to share tracking data with providers.

### Benefits of Tracking, Even Without Provider Involvement

Consistent with prior literature on the value of behavioral self-monitoring [8,39], many physicians considered tracking as an opportunity for patients to learn about their behaviors, symptoms, weight-management progress, or other health outcomes, and generally as a way to manage their health. If patients can track, review, and analyze what they do on a daily basis, it “will change how [they] do it” (participant GM10). Participant D15 described how tracking data becomes an “eye opener” for some of her patients. Tracking helps weight-management patients see if, and how, they need to change their food intake and can help IBS patients identify how certain symptoms correlate with specific foods.

Some physicians noted that as people develop good tracking practices, they become capable of identifying trends and correlations between their behaviors and symptoms, and become more “independent of physicians” (participant GM09). When people in a weight-loss program are already tracking their diet and activity, participant D12 worries less about their chances for success: “...they are a little bit more motivated, they are going to become more educated in that, and that is going to help them meet those goals.”

### Provider-Perceived Patient Barriers to Tracking

Providers also described many barriers that prevent patients from effective tracking. Some tracking requires a regular commitment of time and effort, especially for data that cannot currently be logged automatically and unobtrusively, such as food intake. Therefore, many patients do not adhere to tracking with sufficient frequency or detail to make this data clinically useful. Patients might also not understand what to track and how to track well. Some patients may track the wrong data or may consistently not track well or accurately enough to gain benefits; these patients may lose their motivation for continued tracking.

Patients also sometimes have unrealistic expectations for how quickly they will receive benefits from tracking. They become discouraged when they do not achieve them. Even when patients track regularly and track the right factors, the apps they use do not always help them draw actionable conclusions from this data. For example, some IBS patients may expect to identify specific foods that are symptom triggers, when specific ingredients may be the trigger.

To address these barriers, patients may require alternative tools or guidance from health experts on deciding what to track, as well as how to interpret and act upon the data.

### Benefits of Medical Team-Directed Tracking

Providers find that simply encouraging patients to track the data, and later asking them how it is going, overcomes some motivational barriers to tracking and managing chronic illness. As noted in the previous section, additional coaching on what to track, how to track, and how to review the data can help patients learn to track the right data in the right ways. This can also positively impact patients' confidence in their ability to benefit from tracking. Many providers—physicians, nurses, and dietitians—considered themselves responsible for educating patients on how to track and make use of data:

We play a role of educating patients about the use of the data, so that they don’t overreact or underreact. Putting them into our perspective, and then being able to use that across the board.

Participant N04, who uses symptom diaries

### Benefits of Reviewing Tracked Data

#### Overview

Provider review of patient-collected data can offer a variety of further benefits. Many providers reported using patient-collected life-log data to make, modify, or confirm diagnoses. Others use the data to tailor treatment plans to patients’ routines.

#### Supporting Diagnosis

For providers working with patients in weight-management or chronic disease-management programs, diagnosis and treatment is usually a multi-step and multi-provider process. They rely on patient-collected data across multiple visits to make and adjust their diagnosis and treatment plan. In most weight-management programs, primary care physicians often diagnose overweight or obesity by assessing body mass index (BMI), waist measurement, and other health risk indicators. After diagnosis, patients are often referred to a dietitian for diet and exercise management. Participant D15 described her process during weight-management dietary consultations:

At an initial visit I probably spend 15-20 minutes on reviewing what they eat [using a diet recall] and pen that down. When they bring a food record in we’ll spend about the same amount of time going over that. Sometimes people will send me records then I follow up by emailing them and asking questions. Usually what I’ll do is look at the whole thing to pick typical days and a few other random days so that I can tell the difference between calories.

Participant D15

Participant GM09 described a similar approach for using food and symptom journals with potential IBS patients:

If I see a patient for an initial encounter and if I think that they have IBS, I would give them a task to collect some data then see them back in a couple of months. Then I’ll review it with them and see if together we can come up with some trends or interventions that might be beneficial for them. Once we have a diagnosis and institute a treatment plan then my goal in each visit is to see if that’s working or if we need to adjust our management plan.

Participant GM09

Participant D2 described an experience working with a patient who had trouble determining the reason for her weight increase. After she started tracking her food intake and exercise, participant D2 and the patient reviewed the food log. Together, they found out how many calories came from the bottle of wine the patient drank each day. After identifying and acting on this opportunity for change, the patient began to lose weight.

With multidisciplinary teams becoming increasingly important in primary care, providers also use patient-collected data to support decisions about when to involve other medical team members. Participant FM01 imagined that noticing poor sleep hygiene from a patient’s sleep diary might lead him to refer the patient to a behavioral psychologist to evaluate any psychological etiology for insomnia. Participant D12 also believed that access to patient-collected data by all medical providers allowed for a more cohesive team approach for patient care:

[If patient-collected data indicates that other psychological factors, not just dietary intake, are affecting symptoms] the dietitian gets support too. Because you got other providers working with the patient too.

Participant D12

#### Personalizing Treatment

Reviewing self-monitoring data together also provides opportunities for providers to learn about their patients. Understanding patient preferences and routines helps providers shape care to individual needs. Participant D13 talked about her experience working with people who described dieting their whole lives, but who were still unable to lose weight. By working with them to understand their routines, she was able to tailor the diet suggestions based on their individual diet constraints or cooking habits.

IBS diagnosis and treatment is a complex process that requires excluding other conditions that produce similar symptoms, categorizing patient symptoms, and determining individualized, heterogeneous triggers. It also requires identifying which symptoms a patient most wants to address and what changes he or she is, and is not, willing to make to manage them. To help with this process, health providers want tracking tools that are more flexible and that support tracking the symptoms most important to their patients:

It would be helpful if the patient has another symptom that they think might be related and be able to track that symptom as well as [what they are already tracking].

Participant GM10

Communicating around shared symptom and behavior logs can also help providers learn about their patients’ priorities, both for symptoms and lifestyle.

#### Increasing Motivation and Accountability

Directing patients to track data and coaching them on what to track benefits many patients, but many others continue to struggle with motivation. Providers find that reviewing data with these patients can show them “why it’s valuable for [them] to collect those data” (participant D11), leading to increased patient engagement with the tracking activity, as well as with the overall treatment plan. This is especially the case when patients are unable to draw meaningful conclusions from the data on their own. Going to the effort of collecting data but not having it lead to improved health or a reduction in symptoms is a frustrating, tiring process. Participant D02 emphasized the importance of reviewing data with patients in this situation:

It’s more helpful if you have someone to review it with, because otherwise it might look like “Why am I doing this to myself? I’m just taking all this time for nothing.”

Participant D02

Consistent with Mohr et al [47], providers also felt their review of patient data could increase patient accountability and adherence to the treatment plan. One of participant D12’s patients noted, “I know I have to turn it in so I'm going to eat healthier.”

### Supporting the Patient-Provider Relationship

#### Overview

Reviewing self-monitoring data also helps providers communicate and build relationships with their patients. Effective provider-patient communication supports information exchange and shared decision making, which may increase patient knowledge about their health status and adherence to the treatment [22].

#### Learning About Patients

Many providers use patient-collected data and the review process to learn about their patients, to “get an idea of what’s going on in their life” (participant GM10). While this information can help identify alternative sources or triggers of certain symptoms, it also can reveal unarticulated patient values and goals. Data common in consumer life-log apps, but not in many provider-preferred tools—"What do you do? Do you live by yourself? How long is your commute? Who else lives with you? What kinds of obligations to your time do you have?” (participant D13)—reveal constraints and opportunities for change. Participant FM06 thought she could discover “what the patient cares about” from tracked data and conversations about that data, and then use this information to motivate the patient to stay in the program. For example, participant D20 asked a patient to record the context of eating and found he ate more when he had peer pressure from cousins and friends. Therefore, instead of just telling him to eat less, they brainstormed ways to improve his diet without sacrificing his social life.

#### Facilitating Discussion and Managing Visits

Many providers use patient-collected data to facilitate discussions during visits. If providers have access to the data prior to or at the beginning of the visit, they can plan the visit agenda around patient concerns or have a topic to initiate the conversation. For participant D02, the tracked data is particularly useful when patients do not have, or have trouble articulating, a clear reason for a visit:

If they don’t know what the problem is, I at least have something to look at, and I can identify where to start to ask questions around rather than having a million things I can ask about but not knowing if any is relevant.

Participant D02

Some providers prefer to have the data ahead of time so they can better prepare for the visit, particularly when a patient collects a considerable amount of data between visits:

If I had seen this [report] beforehand, this would be really nice for me to know what she is planning on coming in and what she wants to talk about so it doesn’t catch me by surprise, so I can prepare for it too.

Participant D12

Others felt they would rarely have time to review patient data before a visit, so they preferred to engage with this patient data only during visits.

Some providers use patient journals to facilitate and create a record of conversations during visits. Participant D13 showed us her favorite paper-based food journal (see Figure 1, c) and explained how she uses it to facilitate her conversations with patients. During a visit, she highlights certain columns to emphasize main points of the conversation or for follow-up. She crosses out other columns to alleviate unnecessary patient concerns and to help focus the conversation. Patients can then take the annotated record and use it to reflect on their behavior. This gives patients an artifact that supports their memory of the conversation and can help them journal more efficiently in the future.

Participant BP03 has his patients practice cognitive behavioral therapy at home and send him their thought records before visits. When they are together in the clinic, participant BP03 shares his computer screen with his patients and they review the data together. This helps participant BP03 and his patients better understand each other’s focus and correct any misunderstandings right away.

### Barriers to Using Tracked Data in Clinical Care

#### Overview

Despite the benefits of reviewing patient tracking plans and patient-collected data in clinical care, providers encounter many challenges when they try to use this data in their current practice. Primary care and gastroenterology physicians found it challenging, if not impossible, to review large amounts of data during short visits and they lack incentives to review it outside of office visits. They also questioned whether they have the appropriate expertise to review the data. Therefore, they prefer to delegate reviewing the data to other medical team members, such as dietitians. These providers typically have longer visits with patients and more expertise and experience in identifying problems using life logs, especially food diaries. However, dietitians experience their own time and workflow constraints to reviewing the data, complicated by electronic tools that do not support their needs. In the next sections, we review these barriers and concerns in detail.

#### Lack of Time

Across providers we interviewed, the common clinic appointment in family medicine or gastroenterology lasts 15 to 20 minutes, leaving less than 5 minutes—more often 1 to 2 minutes—for a provider to review self-tracked data. Many are skeptical about what they can meaningfully achieve in that time. For example, participant FM07 said she does not have enough time during a visit to explain what the data means, and so she chooses not to review it at all.

Some physicians we interviewed believe reviewing data between visits is valuable, but they also feel this work is not recognized and thus they cannot allocate much time to it. Participant FM05 reported he could only spend 5 to 10 minutes per day reviewing patient-collected data across all patients (around 20 patients per day). Current workflows and incentive structures pose “a time barrier that discourages me from reviewing” patient-collected data (participant GM09).

Compared to physician visits, the typical patient visit with a dietitian lasts 30 to 60 minutes. This causes many physicians to delegate the review of self-monitoring data to dietitians and focus on other topics during their short visits. Dietitians normally spend 15 to 20 minutes reviewing tracked data—predominantly food journals, sometimes along with a physical activity or symptom diary—with patients and consider it a valuable part of their consultation.

Many dietitians also work with patients on their tracked data outside of clinic visits. Patient portals and other tools for online communication enable dietitians to review data, discuss barriers to tracking, and recommend changes to a treatment plan on an ongoing basis. Many dietitians believe this is the most effective way to help people to manage their diet for either weight management or IBS symptom control; however, they are hesitant to encourage this practice because this work is unbillable and unpaid across hospital systems. Providers are not normally paid for phone calls, emails, or any electronic communication outside of patient visits. Participant BP03 said the incentive structure “has a perverse, mixed message: collect the data but you don’t have time to do it.”

#### Questions About Expertise and Benefits Offered

Most physicians reported that they are often the first person to see patient-collected data, even if they have doubts about their time and expertise to engage with it. Participant GM09 notes that there is often no alternative: “There is no one (to help me review the data). I review the data myself.”

Facing time constraints and a lack of expertise, many providers prefer to refer patients to dietitians, when possible. Dietitians have more expertise reviewing food journals and the review activity is “more in line with the normal interaction” (participant GM09). Participant GM14 shared that even though she has a degree in nutrition she does not consider it her strength to understand food types and nutrients efficiently:

They (dietitians) ask all of those questions and I don’t. I’m not good at that. I could probably muddle my way through it but it wouldn’t be efficient and I wouldn’t get as good information out of it.

Participant GM14

This support, though, is not always available. While participant FM18 used to refer patients to dietitians for food-related concerns, there are currently no dedicated dietitians in the health maintenance organization (1), so he conducts this review himself.

Other physicians, however, find it more effective to review the records themselves rather than to delegate to other providers. Now that he reviews the data himself, participant FM18 feels that, as the only member of the medical team with regular and consistent interaction with patients, he is in the best position to use the data to offer advice and to enhance his relationship with patients. Participant GM19 also used to refer patients to dietitians, but patients rarely followed up with them. Now he reviews patient-collected data and offers feedback himself.

#### Lack of Flexibility

Tailoring tracking activity and a treatment plan for an individual patient is important for both weight management and IBS. However, providers reported that current consumer apps and provider tools, including mobile phone and Web apps, do not provide sufficient flexibility to address the needs of both patients and providers. For example, for some patients it is necessary to track a myriad of factors and symptoms in detail, but others might only need to focus on a certain type of food. According to participant D13, systems that require or encourage patients to track and review more data than necessary often discourage patients from tracking. Participant FM01 said a system should “have the ability for the individual physicians to tailor it to the way they practice,” otherwise it is difficult for providers to integrate it into their clinical workflow.

#### Lack of Standardization

One way patients achieve some flexibility is by choosing among the many apps available in the marketplace. Paradoxically, this creates problems of data standardization when they try to share this data with their health providers. For some health data, such as glucose level, blood pressure, and symptom history, providers use standardized forms or applications. This standard, consistent presentation facilitates their accurate review of data in a short amount of time. However, consumer-oriented life logs often lack a standardized format, or when they do standardize the data, they reduce it to a factor neither the patient nor the health provider cares about (eg, calories for IBS patients). Participant FM16 wanted to use “physical activity as vital signs” in her practice, but found it difficult to compare or to define “activity level” among various types and levels of physical activities.

Some standardized forms for food journaling have been developed for provider use and have been adopted in practice. However, these forms are often difficult for patients to use (cf, Cordeiro et al [48] and Tsai et al [49]) in social settings and to carry around, which leads to incomplete records and recall bias. Participant D15 describes her frustrating experiences with standardized food journals:

We had food records as little booklets...but the problem is, how do we get that back to us? They either had to mail it to us, or they had to come in and drop it off, or, if we were seeing them again they could bring it. Half the time people forget to bring it. So, it became easier to just say, “Okay, why don’t you record it in any format you want and then either send it to me or bring it to me.”

Participant D15

Like participant D15, many providers do not provide patients with standardized forms unless patients request them. However, when patients track using their own paper or electronic diaries, this creates challenges for providers to review this free-form, inconsistently formatted data. Free-form data and consumer app that limits on data export also prevent providers from generating and viewing meaningful, actionable summaries.

While needs vary according to provider goals, providers all need different, typically more summarized, views of the data than do their patients. For example, while many patients ask providers to review exercise logs, most providers have difficulty efficiently interpreting heterogeneous, detailed physical activity logs. They find that having patients verbally summarize the logs is still a more efficient way to gain insights from it.

### Lack of Mechanisms for Sharing With Providers

Many life-log apps do not offer data export features, other than application programming interface (API) access or sharing to social network sites. This creates barriers to integrating this data into provider practices, especially when they want to be able to review it on a day-to-day basis or during patient phone calls. Participant D15 has better experiences sharing data from paper-based diaries, which can be photographed or scanned, than with current mobile phone apps:

[If it’s recorded on paper] I can keep it and look at it. If they email it to me I can go back and reference it. If it’s on somebody’s phone app then I can’t.

Participant D15

While some providers prefer to conduct in-depth reviews of data only during clinic visits, they also report that the mere potential to access the data remotely would help them achieve more benefits from life-log data, such as increased accountability:

[Patients] will not be held accountable for it because there would be no way I could see it before [the] appointment other than [if] they were emailing it in.

Participant D12

As a result, some providers hesitate to encourage patients to use life-log apps and prefer that patients regularly email records or bring paper forms to visits.

## Discussion

### Principal Findings

Providers had varying goals and needs for using and reviewing life-log data (see Table 3). Everyday life logs can support diagnosis, prioritization for follow-up, patient education, patient engagement, and treatment. These data, particularly from provider-directed tracking, are typically used to determine symptom triggers, to guide patient self-monitoring and treatment, and to motivate patient adherence to diagnosis and treatment plans. This set of patient and provider goals, and corresponding design needs, for IBS and weight management is largely consistent with those identified in work with other chronic conditions [24,31,50].

Life-log data, however, also offer more insight into patients' lives and priorities, and thus offer new opportunities to support patient-provider communication, better patient-provider relationships, and more patient-centered treatment plans. These data may include nonhealth data, such as location histories and calendar information, that can help providers learn about patient values, constraints, and goals, as well as help facilitate discussions and help patients and providers manage clinic visits. Currently, this use is almost exclusively at the patient's initiative, and is largely unsupported by current app design.

**Table 3:** Benefits providers achieve with life-log use in clinical care, as reported for irritable bowel syndrome and weight management.

Provider-perceived benefits	Uses of life-log data	Initiators
Diagnosis support	What and when do patients log? What triggers symptoms? Do patients track the right things in the right ways?	Health providers
Treatment design and planning	What are patient routines and priorities? Do patients adhere to treatment plans?	Patients
Provider-patient relationship building	What do patients value in terms of lifestyle choices? What are important aspects in the context of patient health behaviors?	Health providers or patients

Most of these goals are not well supported by currently available commercial apps, and each set of goals drives different design requirements and opportunities. In this section, we discuss how varying provider goals affect tracking activities and needs, and what design opportunities support each.

### Provider and Patient Goals Affect Tracking Needs

#### Overview

Systems supporting the use of patient-collected data in clinical settings should take into account varying provider goals and tracking needs, such as diagnosis, education, motivation, and relationship goals. Standardization of data formats, accompanied by personalization of which data are collected and how they are presented, will help to support these goals while preventing data from overloading providers and patients [51].

The various patient and provider goals (eg, diagnosis, education, agenda setting, and relationship building) require different balances of personalization and standardization.

#### Supporting Diagnosis Versus Supporting Interaction

When developing potential treatment plans, providers look for detailed information during their review of patient self-monitoring data. If their goal is to identify specific triggers for symptoms, they look for correlations between factors, whereas if providers are monitoring a symptom or outcome, they try to identify trends and outliers in the data. For example, when working with patients in weight-management programs, providers need precise portion, nutrient, and calorie information to be able to make diet recommendations. When working with IBS patients, providers focus more on common food triggers for IBS symptoms: lactose, fiber, and fermentable, oligo-, di-, monosaccharides and polyols (FODMAP) foods.

However, when providers use patient-collected data to support their interactions with patients, they often look to understand patient goals and priorities. For example, if a patient’s goal is to walk 10,000 steps per day, the provider may focus on how frequently the patient meets this goal. Then they can start a conversation about the patient’s experience, give suggestions, and discuss remaining barriers. What patients emphasize in symptom logs or food diaries can help providers identify patient priorities and routines about which patients are and are not flexible. Therefore, systems to support patient-provider interactions should provide different granularity of summaries of tracking data that helps providers effectively focus on important facts alongside patients’ subjective experiences of this data.

#### Educating Patients Versus Engaging Patients

Providers play a role in educating patients about their symptoms, health status, tracking, and outcome management. A system can support this educational role. Many traditional telemonitoring systems allow providers to leave feedback on patient-collected symptoms outside of visits [24,33] or to facilitate patients in making sense of their data [31]. Consumer life-log tools have rarely been designed to support this collaborative education, and there are exciting opportunities to do so. For example, automatic synthesis of food journal entries may help providers explain what FODMAP foods are to a newly diagnosed IBS patient, using example foods the patient commonly eats. They could also then discuss categories to focus on, and customize the app to provide feedback on this.

For weight management, many systems support automated calculation of calorie intake and expenditures within a day or over time, helping people see the effects of their choices [49]. These systems could better support patient-provider conversations around diet plans by allowing providers to demonstrate how small differences in choices in the prior weeks could have led to different calorie balances. Such an app could then allow providers to save these simulations as a nutrition plan for the upcoming week.

Providers also use their understanding of patient values, routines, life events, and how tracking fits in with these contextual factors to help keep patients motivated to follow their tracking or treatment plans. For example, participant D13 had an experience when her patient did not fill in the record because her daughter was sick and she did not have time to cook. Seeing this reflected in the record prompted participant D13 to have a supportive conversation to comfort the patient rather than reminding her of the importance of tracking. Therefore, systems to support this provider goal should be capable of collecting and communicating relevant contextual information. While systems to support diagnosis and disease management require high-reliability-tracking technology [52], use of engaging, but perhaps less reliable, consumer-centric tracking apps might offer better support for patient-provider communication around routines, values, and priorities. Prior work (eg, Grönvall and Verdezoto [53]) has noted the importance of routines and values. The ability to use current consumer-centric tools to identify this information, however, has not been previously articulated, at least not in support of some provider goals and for collecting the data; presenting data remains a barrier.

### Limitations of Current Systems and Design Opportunities

#### Overview

Providers who have experience using various tracking tools report that some tools are better at supporting their goals than others. Traditional paper-based diaries still play an important role in many provider-patient interactions, while computer- and mobile phone-based systems provide features to support remote interaction and dynamic representation of data. By understanding the benefits of each system, designers can create life-logging and other self-monitoring apps that will better support provider needs.

#### Dynamic Representation Versus Flexibility

Computer-based systems have the potential to synthesize data automatically and present dynamic summaries. This helps providers and patients identify trends, correlations, and regular or irregular events during the tracking period. However, most current systems do not give providers the flexibility to adjust tracking plans for what to track and how records and summaries are presented; they do not generate summaries in formats that providers need. As a result, patients often spend considerable effort collecting irrelevant information or collecting potentially valuable information that providers never fully review. On the other hand, paper-based records preserve the flexibility for providers to adjust what data a patient tracks and how they view individual records. Without the resources to enter these records into a standardized database, however, it is impossible to generate summaries automatically or to correlate them with other data. When patients are diligent in tracking and they bring this data to visits, providers must quickly “eyeball” the data and summarize it themselves.

Future tracking systems should allow providers and patients to customize the data that they track and the ways in which it is presented. Many telemonitoring systems have been designed to allow patients to transmit tracked data to their remote health care team members in a standard format [54]. However, these systems have been designed primarily with the provider’s objectives in mind, collecting data and health outcomes at intervals that are meaningful and practical to health care providers. These systems rarely integrate with patient-preferred data collection tools—commercial mobile phone apps or wearable devices—and thus create unnecessary barriers for patients to integrate the tracking process in their everyday routines. Some do not even provide interfaces for patients to view their data. When patients cannot benefit from tracking without involving their health providers, it is difficult for them to stay engaged with the tracking activity [55].

Previous efforts to integrate consumer health data flexibly into health providers’ routines have largely failed. Google shut down Health [56] and Microsoft has not reached critical mass with HealthVault [57,58]. Weaknesses included a lack of integration of consumer data into these systems and lack of subsequent use in health care provider workflows [58,59]. Newer frameworks such as Apple HealthKit, Microsoft Health [60], and Google Fit [61] allow interoperability and data portability between different consumer apps and provider tools. They address some of the earlier concerns about poor integration, and may become an important enabling infrastructure by allowing patients and providers to customize tracking tools for an individual based on his/her routines, preferences, and needs while still presenting the data in a consistent, familiar interface for providers.

We caution, however, that while modern health data-sharing systems and frameworks may enable better data sharing for diagnosis and treatment, they are in many regards still less capable than paper-based tracking tools. Their highly standardized formats lose some of the contextual richness that supports patient-provider relationships, communication, and visit management. These formats are also less likely to support the collaborative interactions patients and their providers have around what and how to track using paper journals. For example, while new health frameworks enable consumers to import data from many different tracking tools and share with an app of their provider’s choice for review, this unidirectional process reduces opportunities for providers and patients to collaborate on how to use a tool and which specific data to track.

PatientsLikeMe [62] tries to bridge digital and paper formats by allowing patients to track some health factors digitally and then print Doctor Visit Sheets [63,64]. One-third of respondents in a 2010 survey of PatientsLikeMe users reported using Doctor Visit Sheets to support health care visits [64], but none of the providers in our study had encountered one of these summaries. This suggests that the tracking and summarization in PatientsLikeMe works for some users and some conditions, but not those in our study. Tracking in PatientsLikeMe best supports medication and side-effect tracking, with diet tracking limited to recording vague, nonspecific changes such as “diet modification” for obesity or “low FODMAP diet” for IBS. It also only supports limited integration with other apps or devices, supporting only Fitbit. Similar to modern health data-sharing frameworks, the visit sheets can lose much of the context captured in consumer health and life-logging apps. While this tracking may be useful for understanding the efficacy of medications or broad treatment approaches at the population level, the current capabilities are of limited use to patients seeking to manage IBS, weight, or similar conditions.

#### Supporting Remote and Face-to-Face Interaction

Providers want self-monitoring tools and their organizations to provide better opportunities for patients and providers to communicate and review data in person and remotely. Patients typically bring the data to their office visits, where there are many challenges to reviewing the data together. In many clinic rooms, arrangement of computer screens does not allow both the provider and patient to review the data. When providers review data on their screen or on a borrowed mobile phone, it creates barriers to eye contact and communication similar to the challenges of using a computer to review electronic medical records (cf, Alsos et al [26] and Chen et al [27]).

Providers often need to discuss, review, and ask questions about life-log data in a collaborative fashion, which is different from when they interact with medical records by themselves and then explain the results to patients. Therefore, instead of designing interfaces and rooms to minimize attention to digital devices [26], designers wishing to support life-log use in clinical settings should provide mechanisms to support shared, collaborative review and interpretation. Sharing life-log app data on a mobile phone, as is often necessary when patients use consumer apps, also means that limited information can be displayed at one time. Paper-based diaries give providers and patients the flexibility to lay out and rearrange different pages during face-to-face interactions, but they limit synthesis or remote sharing and review.

Clinic administrators and app designers might explore screen-casting capabilities. Sharing mechanisms have been described in previous research [24] and by providers that we interviewed (participants D02, D15)—patients either bring their own device or share their passwords with providers. In contrast, screen casting would allow patients and providers to review more data on a shared screen and still allow patients to retain “ownership” and control of their data. Collaboratively annotated individual records or summaries would also provide patients with an artifact of the conversation that the patient could use to support their tracking and treatment between visits [24].

#### Organizational Support

Reviewing patient-collected life-log data can help providers manage visits and develop or adjust treatment plans. It can also support patient-provider communication. By helping providers learn about patient values, goals, and constraints, and by offering them real examples from patient data to use in conversations, life-log data supports patient-provider communication, which supports better patient outcomes [21]. On the other hand, health care decision makers report barriers to adopting technology-based tools, such as limited budget and resources or concerns about reimbursement policies [65]. Similar to the providers in our study, primary care teams in other recent studies report lacking the time, education, and compensation necessary for them to review dietary data and provide dietary consultation, creating barriers to adopting electronic-based dietary assessment tools [50]. To achieve the benefits and overcome the barriers associated with provider review of tracking data, policy makers and health system leaders should evaluate whether incentive structures and clinic visit workflows can be revised to enable potential benefits to be achieved more reliably.

Our study provides one of the first in-depth examinations of clinician attitudes toward, and experiences with, using life-log data in clinical care. The perspectives of these front-line providers will help guide the design of life-log tools, support providers and patients with accessing this data, and steer policies regarding integration of both into current processes. Future *in situ* observational studies could provide more-detailed insights into the process of reviewing life-log data and its influence on clinical workflow.

### Conclusions

In this study, we characterized the benefits health providers gain through use of life-log data, as well as the barriers they face in achieving these benefits. In particular, we identified two broad categories of life-log data use in IBS and weight management: supporting diagnosis and treatment, as well as building patient-provider relationships in ways that support open communication and tailoring treatment plans to patient priorities and routines.

Regardless of goals, providers face many barriers to integrating life-log data into current practice. Even though many patients first bring their data to their physicians, physicians prefer to refer these patients to providers with more accommodating workflows and better-matched expertise. Dietitians, on the other hand, have to work with patients outside of clinic time, without extra reimbursement, to make the best use of the data. Current apps hinder effective tracking and data sharing by not offering flexibility to tailor tracking plans, by not supporting standard data types and summaries for provider review, and by not providing a mechanism for provider review on a day-to-day basis.

New health data-sharing frameworks, such as Apple HealthKit, Microsoft Health, and Google Fit, promise to integrate consumer-collected life-log data automatically, including data sensed by the companies’ own platforms. Apple has worked with the Mayo Clinic and other leading health care organizations to design for integration and summarization from the start [66], but these improvements may not be enough to ensure successful adoption. Designers of health apps should be aware of the range of benefits life-log data can offer, and of the different fidelity and presentation requirements for each goal.

## Appendix

Multimedia Appendix 1 Semistructured interview protocol.

Multimedia Appendix 2 Affinity diagram labels.

## Abbreviations

AHRQ: Agency for Healthcare Research and Quality
API: application programming interface
BMI: body mass index
EHR: electronic health record
FODMAP: fermentable, oligo-, di-, monosaccharides and polyols
IBS: irritable bowel syndrome
NSF: National Science Foundation
