# Supplementary material for: More Than Telemonitoring: Health Provider Use and Nonuse of Life-Log Data in Irritable Bowel Syndrome and Weight Management
Source: J Med Internet Res. 2015 Aug 21;17(8):e203. doi: 10.2196/jmir.4364 (PMC4642406; doi:10.2196/jmir.4364)
Supplement: Multimedia Appendix 1 [file jmir_v17i8e203_app1.pdf]

Appendix 1 – Semi-structured interview protocol

Have you ever reviewed any patient-collected data during patient visits?

1. *[If yes]* Could you please tell me about one experience -- maybe your best experience, maybe your worst, or just the most memorable -- when you worked with patient-collected data?
    a. What condition or conditions were you focusing on?
    b. What data did you work with?
    c. Did you ask them to collect it, or did they bring the data in on their own?
    d. [*If the provider asked the patient to collect it:*]
        i. What caused you to make that suggestion or recommendation?
        ii. How did you instruct the patient to collect this data?
        iii. Did you provide them with any written instructions or pre-formatted record sheets?
        iv. How much time did you spend explaining how to collect this data?
        v. Did you plan to review it, or just for the patients to collect it and review it themselves?
    e. [*If the provider asked the patient to collect it:*]
        i. How did their collection and your review of the data come up?
        ii. What was your reaction at first?
        iii. What was your reaction afterward?
    f. How was the data collected (i.e. mobile app, paper diary, excel sheet, handout or form)?
    g. How did you review that data?
        i. Do you think it supported any of your goals for providing patient care? *Probe:* What? How?
        ii. Did you put this into the patient's medical record?
        iii. What helped it go well?
    h. What could have helped it go better?
2. Now, thinking about your experiences in general, how often do you review this data when you work with your patients?
    a. *Unless it is never or almost never:*
        i. Could you please describe your best experience with using patient collected data?
        ii. And how about your worst experience?
        iii. What conditions or diseases did you find patient-collected data the most useful for? The least useful for?
    b. *[If never or almost never]:*
        i. Why not? *[probe for what might reduce those barriers]*
        ii. Do patients ever ask you to review their data, without you suggesting it? *[probe for what they bring and why]*
        iii. Have you ever not reviewed a patient's data they brought in with them? Why not?

Next, I'd like to step back and ask some about how your use of this patient collected data fits -- or doesn't fit -- into your workflow and what you need as a medical provider.

1. Can you walk me through your typical workflow for a clinic visit? [Both during a visit and outside of a visit]
2. What is your goal for a patient visit? What is your overall goal for a patient across multiple visits?
3. How do you foresee the patient-collected data would best fit into this workflow? When should it be reviewed?
4. How much time do you realistically think you can or would you be willing to spend reviewing the data? Why?
    a. Would you do this before, during or after the clinic appointment?
5. Are there enough incentives for you at this time to review patient-collected data? Is it worth the trouble? [probe to understand values, constraints, etc. For each, ask "why?"]

    a. What would give you the incentives to review patient-collected data? (i.e. patient clinical improvement, ability to bill for its review, less patient phone calls, time saved in clinic, decreased healthcare utilization)
6. Who do you think should review the data? Why?
    a. Would you consider hiring or allocating time to medical staff to review patient-collected data if it would add value to patient care?

For the following questions, we would like to understand your perceived value and adverse effects of patient-collected data even if you don't review them right now.

1. What are the potential benefits of having patients self-collect data? What are the potential downfalls of having patients self-collect data?
2. What are the potential benefits of patient-collected data to the provider? What are the potential downfalls of patient-collected data to the provider?
3. Do you think patient-collected data could still be valuable to patients even if it was not shared with their medical providers? In other words, does a provider always need to be involved reviewing self-collected data for medical care? [Probe: What would be your main contribution (or another provider's) in reviewing patient-collected data? Probe for what benefits they get now or which ones are hypothetical.]
4. What are the collective potential benefits in the patient-doctor relationship? [probe to understand values, constraints, etc. For each, ask "why?"]

What can make certain patient-collected data challenging to review or not useful?

If the difficulties you mentioned were removed, what medical conditions and/or patient population would most benefit from patient-collected data?

1. What type of data would you want patients to collect?
2. How would you use them?
3. What would you want to see?
4. What would you look for in their data?

We're particularly interested in [weight management | IBS].

1. What, if any data, do you you currently use for [weight management | IBS]?
2. What patient-collected data do you think might be helpful for [weight management | IBS]?

Now, I'd like to show you some example output from lifelog applications, each with some hypothetical patient data. I'll describe each and then ask you some questions about it. . As you answer the questions, you are welcome to draw or write on each example interface.

1. These interfaces included views of some data that it sounds like you don't currently use -- such as *[list based on what they talked and didn't talk about]*. I'm curious for what, if anything do you think these might be useful for you to review? *[probe work through them one at a time]*
    a. At what stage would provider input be valuable - suggest to collect, review, both, etc?
    b. For whom?
    c. What would it take for them to be useful?
