# Supplementary material for: More Than Telemonitoring: Health Provider Use and Nonuse of Life-Log Data in Irritable Bowel Syndrome and Weight Management
Source: J Med Internet Res. 2015 Aug 21;17(8):e203. doi: 10.2196/jmir.4364 (PMC4642406; doi:10.2196/jmir.4364)
Supplement: Multimedia Appendix 2 [file jmir_v17i8e203_app2.pdf]

Appendix 2 – Affinity Diagram Labels

Note: We emphasize that the affinity diagram was a tool for the research team. It is not intended to represent a summary of results.

Patient benefits and barriers of self-tracking data

**Level 2**	**Level 3**
Patients gain benefits by tracking themselves and do not necessarily need to review with providers	Tracking help my patients learn
	The act of tracking can be therapeutic
	Tracking with doctors can help motivation
	I'm not sure there is value in me reviewing the data
	How I think patients should see data
Currently there are multiple ways of tracking, some of them are more recommended than others	How patients track
	Special tracking variables
Patients may not want to track because of the workload and the financial burden	Tracking is too much work for patients
	My patients are not interested
	Insurance doesn't cover the consultation
	Current methods are not useful
If patients don't have enough skill using technology or understanding medical terms, tracking is difficult.	Older patients have a harder time with technology
	Not everyone has sufficient tech literacy or access
	My patients are not familiar with tracking
	My patients have medical literacy problems
	My patients don't know enough to track well

Benefit of review self-tracking data

**Level 2**	**Level 3**
When providers review data, they look for information that helps to make diagnoses	Correlations between factors are important
	Seeing trend/outlier is important
	Graphs help me see patterns or trends
	Tracking is useful for these disease/symptoms
	I need some sort of automatic synthesis of the data
	I want to aggregate my patients to find larger trends
	Diagnosis is a multistep process
	Dietary intervention works better than medication
Reviewing tracking data helps providers to gather information from patients	Recall is not truth-worthy
	Viewing data together help with discussion (It helps us focus)
	Use data to answer my question to patients
	I like how self-tracked data help me learn about my patients
	Tracking shows me my patient is committed to be part of the solution
	Patients need to work hard/detail enough to make reviewing worthwhile
Reviewing data helps providers better interact with patients	I want to take account of emotional context of data
	I want contextual data
Reviewing data helps providers manage their visit and interaction between visit	I want remote interaction with patient (I want the data link with EMR)
	How I manage my patient visits
Reviewing tracking data helps patients gather information from providers to manage their health	Having me look at their data & listen can be therapeutic
	Treatment involves a team of providers
	I want to identify patient barriers of tracking and managing their health issues
	Review tracking help my patients learn

Barriers of review self-tracking data

Level 2	Level 3
Providers have difficulty integrating reviewing data into their workflow	I don't get paid to do this
	I have very limited time (I need time to prepare before reviewing data with patients)
	Someone else should do this
	Experience reviewing tracking data matters
	I don't use individual data for prescriptions
	I don't know how to interpret the data
How to track and what to track needs to be personalized	I want to customize treatment
	I think tracking works best combined with goal-setting
	Some tracking is ongoing, some is one time
	Some patients are experts
	Not every tool is right for every patient - I need different ones/flexibility
Some providers have doubts about using tracking or reviewing tracking	How do I track compliance?
	Medication works better than dietary intervention
	I don't trust the system
	I want more evidence to believe tracking/reviewing tracking work
Providers needs more simple presentation and interaction with data	The screen is too small
	Fewer variables make it easy to understand
	I can't deal with words
	The record is too detailed
